# Metabolomics insights into the interaction between *Pseudomonas plecoglossicida* and *Epinephelus coioides*

**DOI:** 10.1038/s41598-022-17387-6

**Published:** 2022-08-03

**Authors:** Jun Zeng, Zhiqiang Yang, Yue Zhong, Yingli Zheng, Jingwen Hao, Gang Luo, Qingpi Yan

**Affiliations:** 1 College of Ocean Food and Biological Engineering, Jimei University, Xiamen, 361021 China; 2 Fisheries College, Jimei University, Xiamen, 361021 China; 3 Xiamen Key Laboratory of Marine Functional Food, Xiamen, 361021 China

**Keywords:** Ichthyology, Metabolomics, Bacterial host response

## Abstract

As a highly infectious epidemic in aquaculture, *Pseudomonas plecoglossicida* infection results in high mortality of teleosts and serious economic losses. Host–pathogen interactions shape the outcome of an infection, yet we still understand little about the molecular mechanism of these pathogen-mediated processes. Here, a *P. plecoglossicida* strain (NZBD9) and *Epinephelus coioides* were investigated as a model system to characterize pathogen-induced host metabolic remodeling over the course of infection. We present a non-targeted metabolomics profiling of *E. coioides* spleens from uninfected *E. coioides* and those infected with wild-type and *clpV*-RNA interference (RNAi) strains. The most significant changes of *E. coioides* upon infection were associated with amino acids, lysophospatidylcholines, and unsaturated fatty acids, involving disturbances in host nutritional utilization and immune responses. Dihydrosphingosine and fatty acid 16:2 were screened as potential biomarkers for assessing *P. plecoglossicida* infection. The silencing of the *P. plecoglossicida clpV* gene significantly recovered the lipid metabolism of infected *E. coioides*. This comprehensive metabolomics study provides novel insights into how *P. plecoglossicida* shape host metabolism to support their survival and replication and highlights the potential of the virulence gene *clpV* in the treatment of *P. plecoglossicida* infection in aquaculture.

## Introduction

In aquaculture, the ‘visceral white spot disease’ of teleosts is associated with the infection of a Gram-negative pathogen of *Pseudomonas plecoglossicida*^1^*.* As a highly infectious epidemic, the infection of *P. plecoglossicida* can cause typical symptoms of numerous white nodules in the spleen, kidney or liver and particularly high mortality in teleosts, resulting in serious economic losses for aquaculture^2^.

Due to considerable harm to aquaculture, the complex interaction between *P. plecoglossicida* and host fishes was increasingly noted. In our previous gene expression studies, *P. plecoglossicida clpV* was identified as a key virulence gene that contributes to the pathogenicity of *P. plecoglossicida* in both *Epinephelus coioides* and *Larimichthys crocea* infections^2,3^. The ClpV plays an important role in the reassembly of the type VI secretion system (T6SS) machinery, as well as the secretion of T6SS effector proteins^4^. The T6SS is molecular machine in the secretion of virulence factors, which can directly inject pathogenic effectors into the host cells^5^. Then, compared with the counterparts infected by the wild-type strain, host fish injected with the *clpV*-RNA interference (RNAi) strain exhibited a significant improvement in mortality and symptoms^2,3^. RNAi of *clpV* resulted in the downregulation of genes in the flagella assembly pathway and a lower pathogen load in host tissues as well as a weaker immune response of the host^3^. In addition, the diguanylate cyclase gene (L321_RS15240)^6^, ABC transporter gene (L321_23611)^7^, secY^8^, pvdE^9^, etc., are also virulence candidates for *P. plecoglossicida* pathogenicity. Although a series of virulence genes associated with *P. plecoglossicida* pathogenicity continue to be discovered, the mechanism of their actions underlying the complex host–pathogen interactions remains obscure thus far.

Understanding host–pathogen interactions is critical for resolving the outcome of an infection. The host senses the presence of the pathogen through recognition of pathogen-associated molecular patterns, while the pathogen evolves numerous strategies to circumvent host defenses and exploit the host cellular machinery^10^. Unlike genes and proteins, the functions of which are subject to epigenetic regulation and posttranslational modifications, respectively, metabolites serve as direct signatures of biochemical activity and tightly correlate with phenotype^11^. These properties make the host cellular metabolome an attractive target for pathogens to introduce phenotypic perturbations that facilitate their survival and replication^11^. Indeed, there is fierce competition for metabolic precursors between host and pathogen, which may alter both pathogen growth and the nature of the immune response^12^. The significant alteration in the host metabolome, involving modulation of glucose, fatty acids (FAs), and amino acids, has been emphasized in recent studies regarding host–pathogen interactions^10,13^. For example, the amino acids arginine (Arg)^13–15^, asparagine (Asn)^13,15^, and tryptophan (Trp)^13,15^ were highlighted as central points of competition between the host and pathogen^13^. Amino acids affect immune responses of the host against a pathogen, such as the function of innate immune cells (e.g., macrophages), the activation and differentiation of T cells and the production of antibodies by B cells^15^. In addition, amino acids influence the physiology and virulence of pathogens^15^. Thus, elucidation of these pathogen-induced metabolic changes would provide novel insight into therapeutic approaches to manipulate and prevent progression of an infection. Nevertheless, the exploration of metabolome disturbances associated with ‘visceral white spot disease’ caused by *P. plecoglossicida* infection has been limited until now.

The emergence of high-throughput metabolomics techniques enables the description of global changes in the host metabolome at the level of individual molecular metabolite species and the identification of metabolite biomarkers^16^. In this study, to identify metabolome disturbances associated with ‘visceral white spot disease’ caused by *P. plecoglossicida* infection, a time series spleen sample set from *E. coioides* injected with PBS, the wild-type strain and the *clpV*-RNAi strain was collected over the course of infection. We performed metabolomics analysis on this sample set using ultra-high-performance liquid chromatography coupled to mass spectrometry (UPLC-MS). As described in our previous studies^2,6–9^, the *P. plecoglossicida–E. coioides* infection model has been successfully established and confirmed to be a suitable animal model for studying the pathogenic mechanism of *P. plecoglossicida* in the laboratory. Considering the highest burdens of *P. plecoglossicida*, the spleen was analyzed in our studies as the representative target organ^2,8^. Then, the comprehensive rewiring of host metabolic networks was investigated over the course of infection to elucidate the potential mechanism of host–pathogen interactions and evaluate the response of *E. coioides* to the key virulence gene *clpV* from the novel perspective of the metabolome.

## Results

### Characteristics of *E. coioides* after infection

According to our previous modeling, which traced mortality and symptoms of *E. coioides* and the abundance of strain over the course of infection^2,6–8^, this *P. plecoglossicida–E. coioides* infection pattern could be divided into two stages, namely, the developing stage (0–72 hour post injection (hpi)) and the terminal stage (after 72–96 hpi). The dynamic distribution of wild-type *P. plecoglossicida* in *E. coioides* showed that *P. plecoglossicida* yielded the greatest abundance in most organs and blood of *E. coioides* at 72–96 hpi^6^. The first mortalities of *E. coioides* infected by the wild-type strain were at 72 hpi, and the majority of deaths were at 96 hpi. Those *E. coioides* presented typical symptoms with numerous white splenic nodules at 96 hpi. Compared with the counterparts infected by the wild-type strain, *E. coioides* injected with the *clpV*-RNAi strain exhibited a significant improvement in mortality and symptoms. No deaths were observed up to 20 days post injection (dpi) for *E. coioides* infected by the *clpV*-RNAi strain. Their spleens failed to develop visible nodules until 5–8 dpi, with the swelling gradually disappearing.

**Figure 1:**
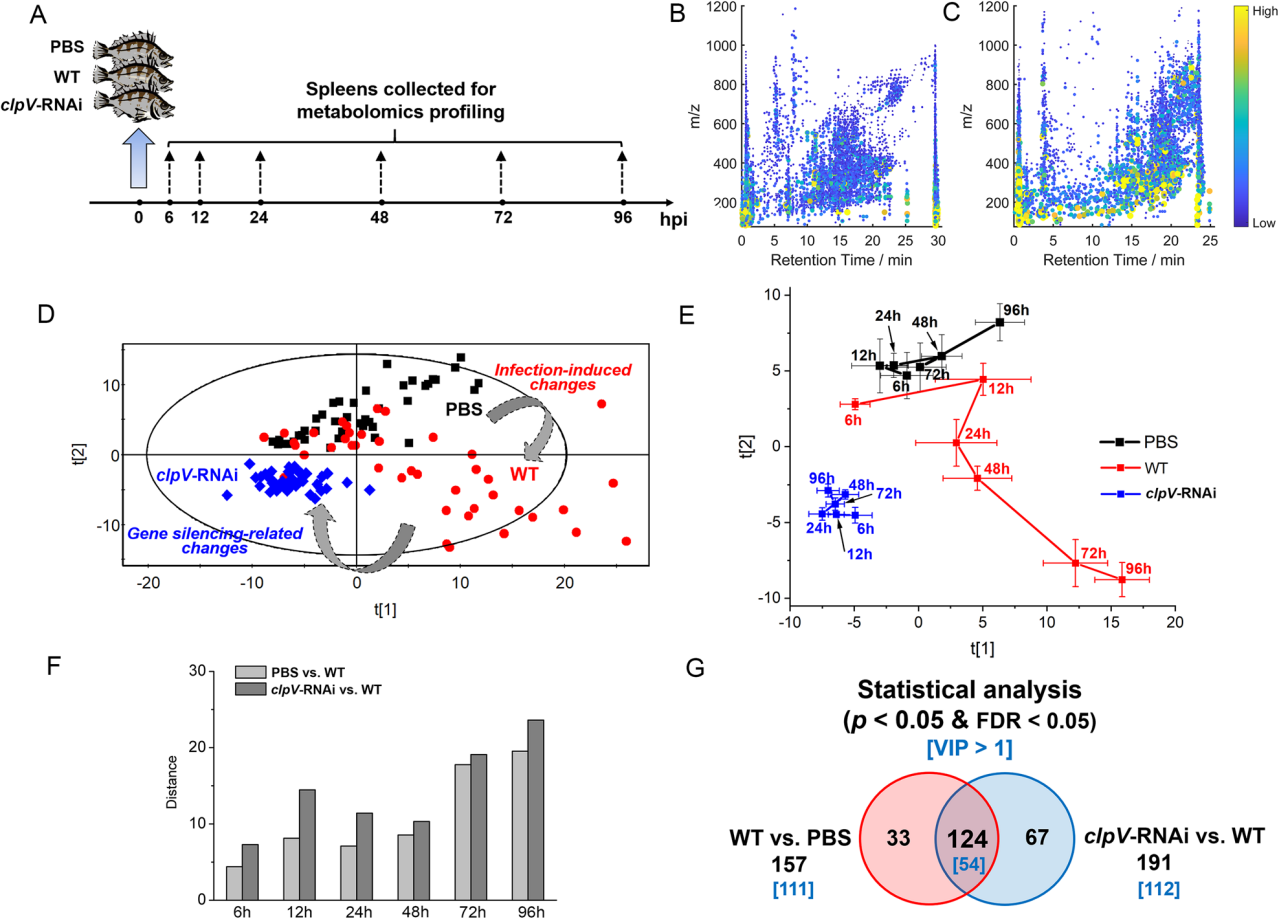
Characteristics of *E. coioides* upon infection. (**A**) Scheme of the experimental design. (**B,C**) Typical metabolite features of pooled tissue extract acquired using LC–MS in ESI positive mode and negative mode, respectively. (**D**) PCA score plot of the metabolic profile composed of metabolites after UV scaling pretreatment. (**E**) Metabolic trajectories of the control and different infection groups based on the PCA model. Each point represents the average score values of samples with SEM. (**F**) Euclidean distance between WT and time-matched PBS (or *clpV*-RNAi). (**G**) Venn diagram for overview of univariate statistical analysis and multivariate VIP values. PBS denotes the PBS control group, WT denotes the wild-type strain infection group, and *clpV*-RNAi denotes the *clpV*-RNAi strain infection group.

In this study, to explore the molecular mechanisms underlying the complex host–pathogen interaction, time series spleen samples from *E. coioides* injected with phosphate-buffered saline (PBS group), the wild-type strain (WT group) and the *clpV*-RNAi strain (*clpV*-RNAi group), were collected over the course of infection (i.e., 6, 12, 24, 48, 72 and 96 hpi, Fig. 1A). Typical histological microphotographs of spleens are shown in Fig. S1. Compared with the PBS group, the spleens of WT individuals exhibited characteristic histological changes at 72–96 hpi. PBS and *clpV*-RNAi groups had considerable histological similarity. These histological sections were consistent with our observation of mortality and symptoms, highlighting the effective role of *clpV* silencing in the improvement of *E. coioides* infection.

### Metabolome of *E. coioides* spleens

This time series sample set was evaluated by nontargeted metabolomics analysis. The large-scale metabolome profiling of *E. coioides* spleens revealed approximately 5000 metabolic features for each analysis mode (Fig. 1B,C). A total of 262 metabolites from a wide spectrum of categories were finally identified, involving 45 amino acids, peptides and analogs (10 essential amino acids, 5 non-essential amino acids and 30 analogs), 9 amines, 4 carbohydrates and carbohydrate conjugates, 18 nucleosides, nucleotides and analogs, 13 organic acids and derivatives, 3 alcohols and polyols, and 170 lipids and lipid-like molecules (12 lipid-like molecules, 4 bile acids and derivatives, 11 fatty acyl carnitines, 10 fatty amides, 48 FAs, 75 glycerophospholipids, 6 sphingolipids and 4 glycerolipids). This profiling unveiled a large diversity and complexity of *E. coioides* splenic metabolome regarding their chemical structures, compositions and polarities. Detailed information on these identified metabolites is listed in Table S1.

The analytical performance of this metabolomics profiling was examined by evaluating quality control (QC) samples and was satisfactory for complex biological samples (Fig. S2). Detailed results of QC evaluation are described in the Supporting Information.

### Global profiling of metabolic disturbance

Non-supervised principal component analysis (PCA) was first employed to gain a global overview of the metabolome changes (Fig. 1D). Two types of metabolic disturbance, infection-induced (i.e., PBS vs. WT) and gene silencing-related (i.e., *clpV*-RNAi vs. WT) changes, were clearly visible on this score scatter plot, as demonstrated by an obvious separation trend among the PBS, WT and *clpV*-RNAi groups (Fig. 1D). It is clear that individuals in the WT group were more discrete than those in the PBS and *clpV*-RNAi groups in this PCA profile, implying severe metabolic deregulation of *E. coioides* upon wild-type strain infection.

The metabolic trajectories of the PBS, WT and *clpV-RNAi* groups were further depicted based on this PCA score plot (Fig. 1E). The euclidean distance between metabolic trajectories at each time point was analyzed to quantify those two types of metabolic differences over the time course. As shown in Fig. 1F, the disturbance of gene silencing would be more potent than strain infection. Metabolic homeostasis of *E. coioides* spleen showed interference beginning at the early infection stage of 6 hpi. Both disturbances caused by infection and *clpV* silencing were noted in the form of more evident metabolic changes at the later infection stage of 72–96 hpi, confirming the discoveries of the *E. coioides* phenotype (incl. mortality, symptoms and histological examinations).

Based on the metabolic profile across the infection stage, we next determined the significantly disrupted metabolites. Univariate statistical evaluation revealed a total of 157 and 191 metabolites that exhibited significant quantitative changes when PBS and *clpV*-RNAi were, respectively, compared with WT (*p* < 0.05 and FDR < 0.05, Fig. 1G). Of note, 124 statistically significant differential metabolites were found in the overlap between these two comparisons. Approximately 80% of significant differential metabolites identified by wild-type strain infection (i.e., PBS vs. WT) showed significant changes by *clpV* silencing (i.e., *clpV*-RNAi vs. WT), implying the efficiency of the key virulence gene *clpV* in this host–pathogen interaction.

Furthermore, two partial least squares discriminant analysis (PLS-DA) models (i.e., PBS vs. WT and *clpV*-RNAi vs. WT) were developed to evaluate these significant differential metabolites based on variable importance in the projection (VIP) values (Figs. S3, S4). A total of 111 out of 157, and 112 out of 191 significant differential metabolites were identified with VIP > 1 when PBS and *clpV-*RNAi were compared with WT, respectively (Fig. 1G). Then, we defined the union of 169 metabolites to be the representative differential metabolites with significant disturbance (*p* < 0.05 FDR < 0.05 and VIP > 1, Table S2).

### Comparison between infection-induced and gene silencing-related changes

**Figure 2:**
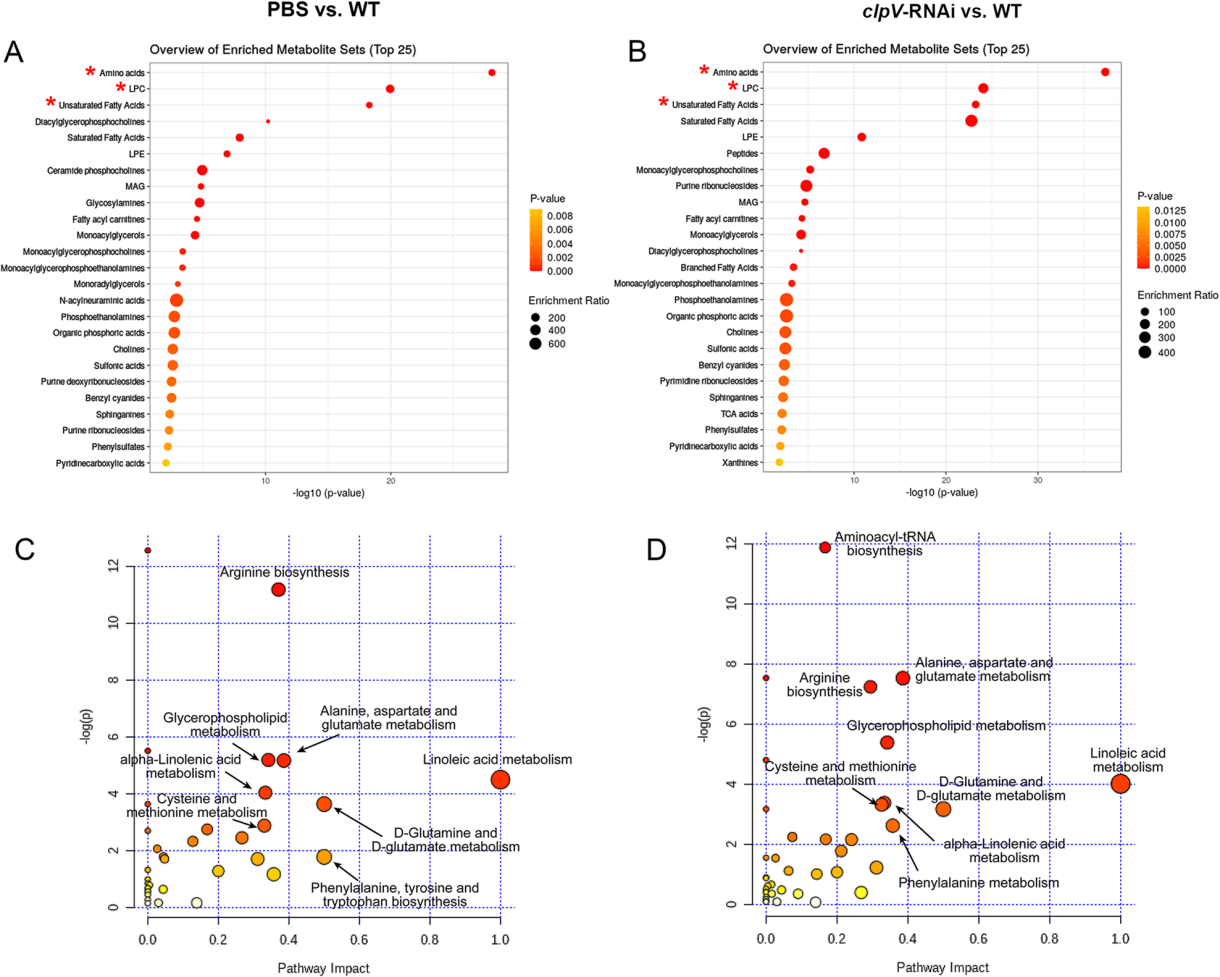
Comparison between infection-induced and gene silencing-related changes. (**A,B**) Overview of enriched metabolite sets. (**C,D**) Summary of enriched metabolic pathways. Enriched analyses of all significant differential metabolites were performed based on comparisons between PBS and WT (i.e., infection-induced changes, left panel), and *clpV*-RNAi and WT (i.e., silencing-related changes, right panel). Analyses of those significant differential metabolites resulted in the top 3 significant metabolite sets (red asterisk*), and revealed the global metabolic disorders of the most relevant pathways.

As identified when PBS and *clpV*-RNAi were compared with the ‘positive’ WT, both enrichment analyses of significant differential metabolites resulted in the top 3 significant terms associated with amino acids, lysophospatidylcholines (LPCs), and unsaturated FAs (Fig. 2A,B). Most of the top 25 enriched disturbed categories were similar between these two subsets of significant differential metabolites (Fig. 2A,B).

Linoleic acid metabolism, arginine biosynthesis, alanine, aspartate and glutamate metabolism, glycerophospholipid metabolism, d-glutamine and d-glutamate metabolism, etc., were regarded to be highly responsible for both comparisons (i.e., PBS vs. WT and *clpV*-RNAi vs. WT, Fig. 2C,D). The similarity in both the most disturbed metabolite sets and metabolic pathways confirmed the prominent role of *P. plecoglossicida clpV* in the host–pathogen interaction.

**Figure 3:**
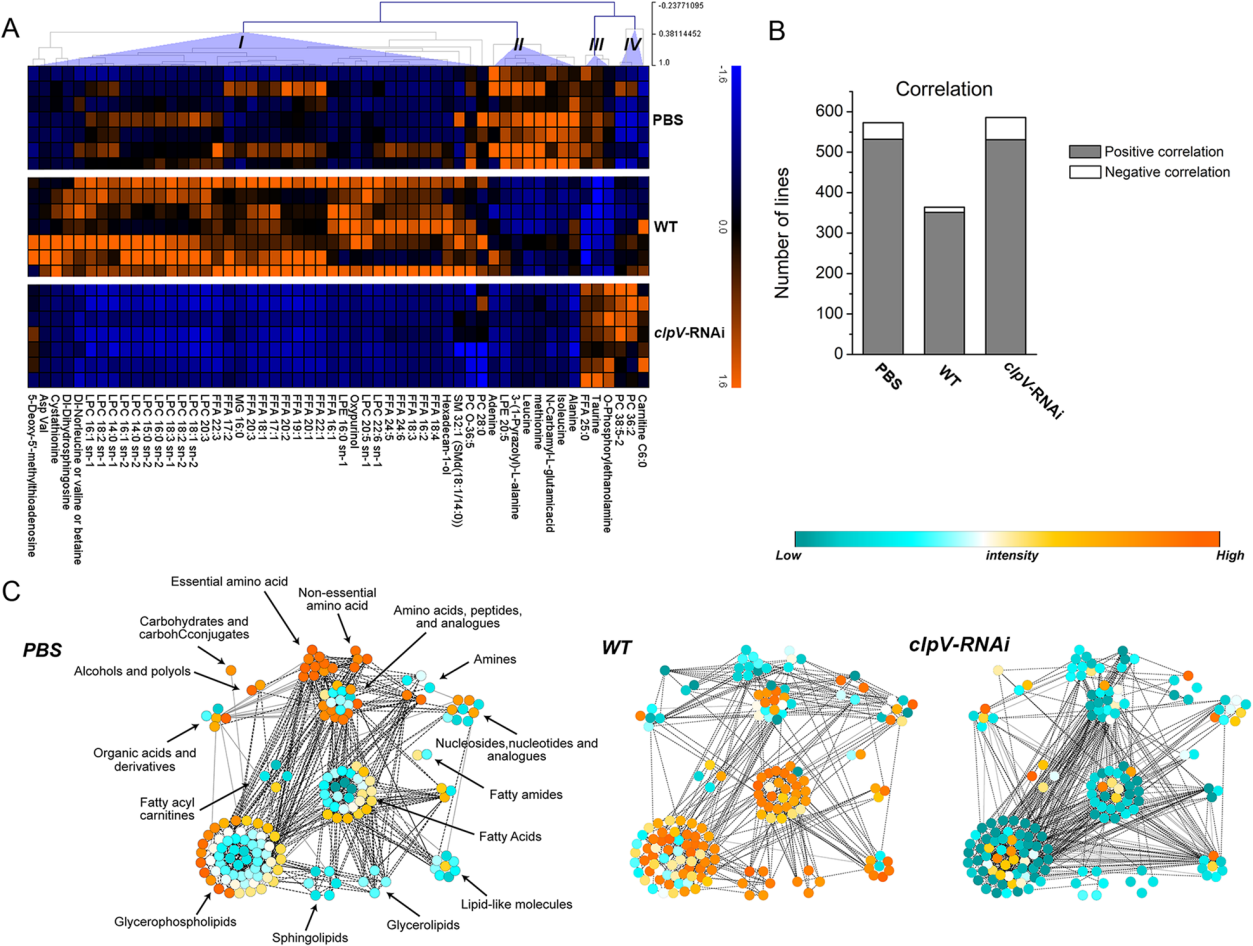
Response patterns of the metabolome at the terminal stage of 96 hpi. (**A**) Heatmap. The intersection of representative differential metabolites (*p* < 0.05, FDR < 0.05 and VIP > 1) from both comparisons (i.e., PBS vs. WT and *clpV*-RNAi vs. WT) was UV-scaled and subjected to hierarchical clustering. The union of all representative differential metabolites was further specialized by correlation networks. (**B**) The number of lines in each correlation network. (**C**) Correlation networks. In correlation networks, each point represents one metabolite with the relative content of UV scaled. Each black dotted (or gray solid) line represents a positive (or negative) correlation with |Cij|> 0.9.

These representative differential metabolites were UV-scaled and subjected to hierarchical clustering and were classified into four major groups according to the response pattern (Fig. 3A). In addition to the significant difference between the ‘positive’ WT and ‘control’ groups (i.e., PBS and *clpV*-RNAi), disparities between PBS and *clpV*-RNAi also existed due to the complexity of host–pathogen interactions. The change pattern was further specialized, as depicted by correlation networks (Fig. 3B,C). Each point indicates one representative differential metabolite with UV scaling, while the line indicates the correlation with |Cij|> 0.9. We observed a decreased correlation between metabolites (i.e., the number of ‘lines’) in WT, while a reactivated coordination of the metabolome was observed in *clpV*-RNAi (Fig. 3B). From the perspective of ‘point’, different classes of metabolites exhibited different changing patterns. Most lipids, including FAs and glycerophospholipids (e.g., LPCs) from Group I in the heatmap, sphingolipids and glycerolipids, had a greater response in WT when compared with PBS and *clpV*-RNAi. Meanwhile, polar and moderately polar metabolites, including amino acids, carbohydrate and carbohydrate conjugates, alcohols and polyols, and organic acids and derivatives, featured a similar decrease after infection (incl. WT and *clpV*-RNAi). Taken together, the silencing of the *P. plecoglossicida clpV* gene improved the lipid metabolism of infected *E. coioides* more significantly.

**Figure 4:**
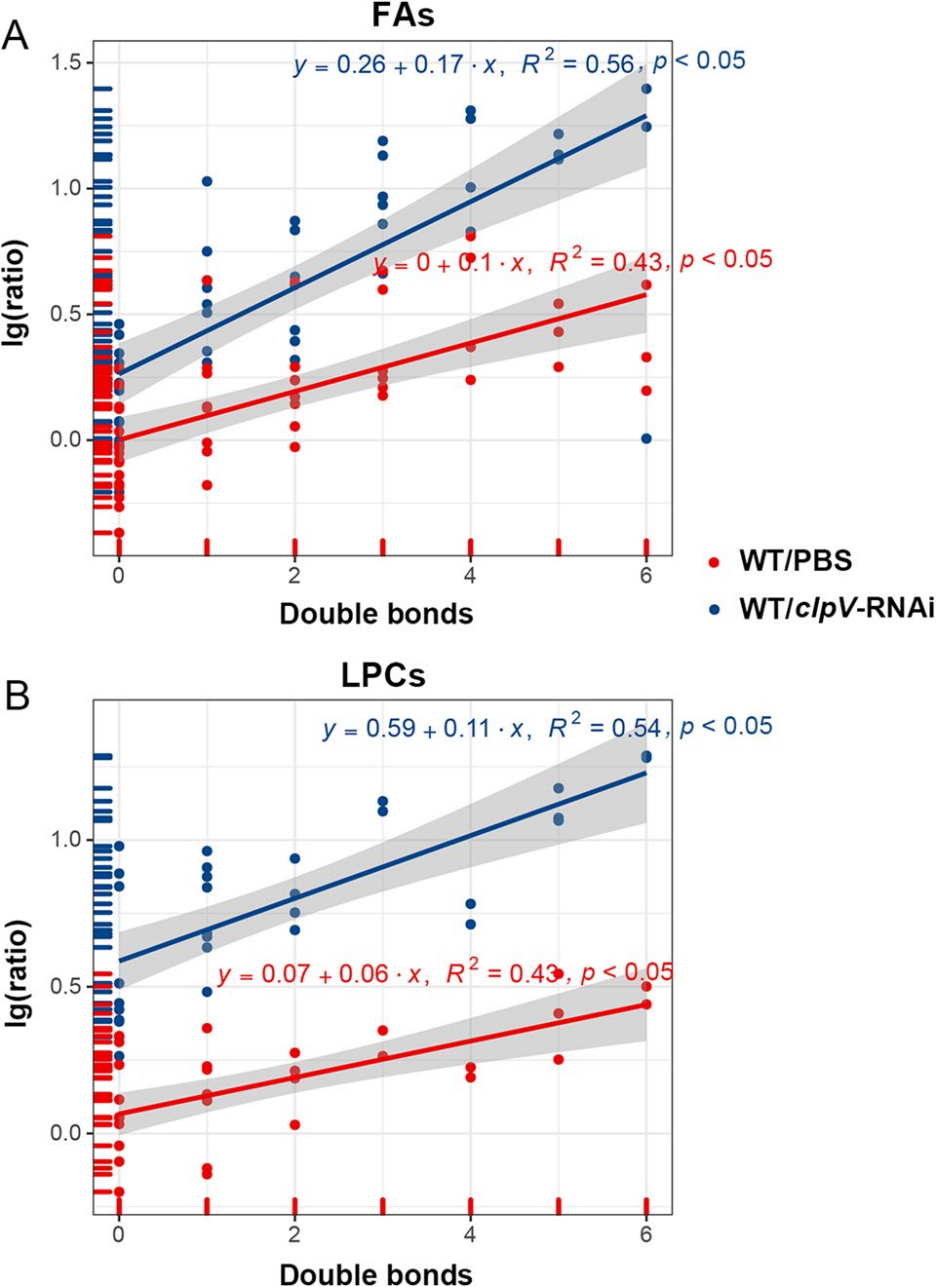
Correlation between response increase in WT to PBS ratio (or WT to *clpV*-RNAi ratio) and the number of double bonds. (**A,B**) Depict the changes in FAs and LPCs, respectively, at the terminal stage of 96 hpi.

In addition to the analysis of response patterns, we further defined whether there were any alterations associated with the chemical structure (Fig. 4). LPCs and FAs, which were defined as the top significant differential metabolite sets in enrichment analyses, had a greater response and higher degree of unsaturation when positive WT were compared with ‘control’ groups (i.e., PBS and *clpV*-RNAi). We found a significant positive correlation between the increase in the WT to PBS ratio and the number of double bonds (*p* < 0.05, R2 = 0.43 for both FAs and LPCs). Meanwhile, their increase in the WT to *clpV*-RNAi ratio had a similar significant positive correlation with the degree of unsaturation (*p* < 0.05, R2 = 0.56 for FAs and R2 = 0.54 for LPCs).

### Discovery of potential biomarkers

**Figure 5:**
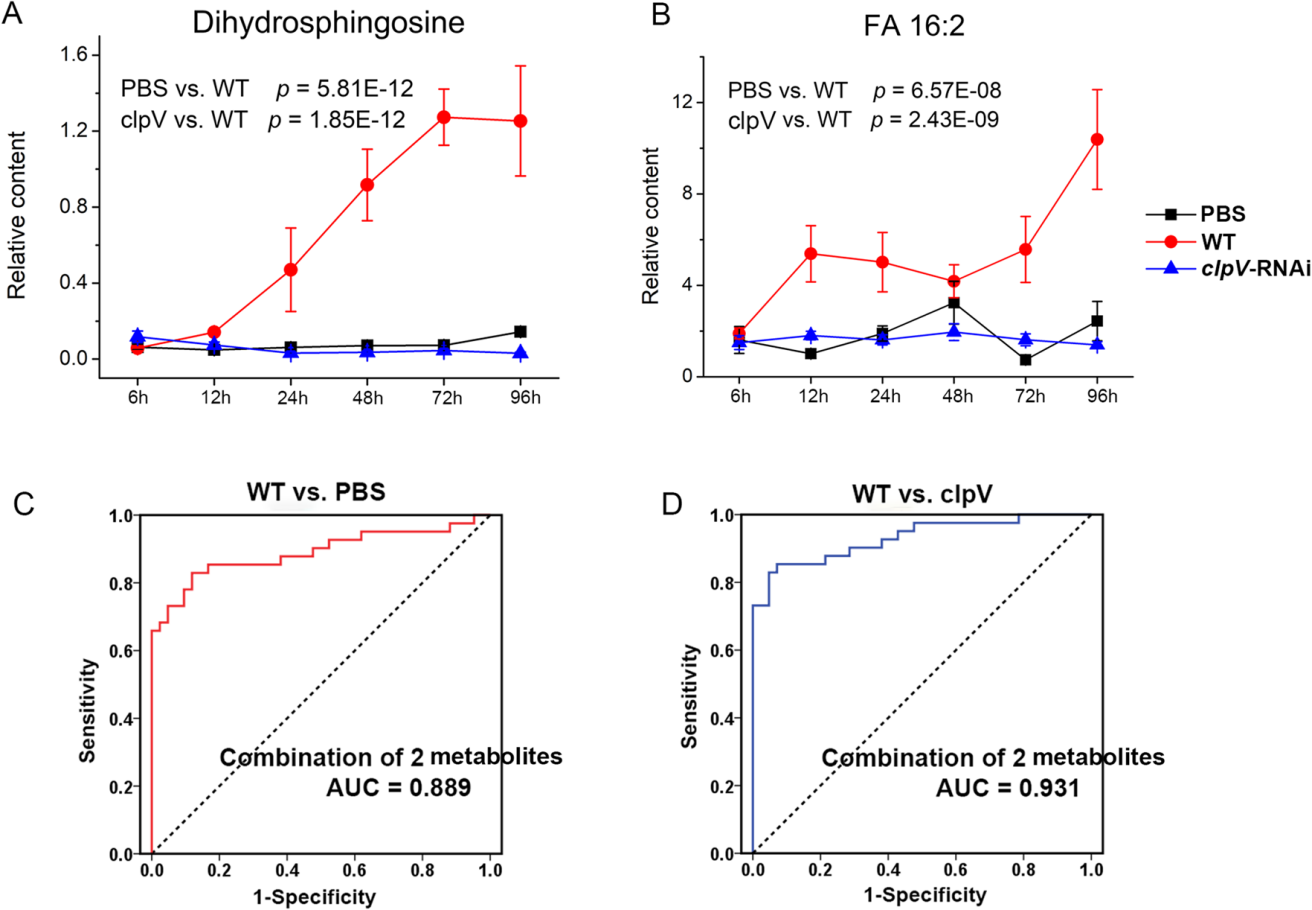
Evaluation of potential biomarkers. (**A,B**) Are the response trajectories of dihydrosphingosine and FA 16:2, respectively. Each point in the trajectory is presented as the mean ± SE. (**C,D**) ROC curves for the combination of biomarkers of dihydrosphingosine and FA 16:2. Diagnostic potential was evaluated based on binary logistic regression.

Potential biomarkers of *E. coioides* that are sensitive to *P. plecoglossicida* infection were analyzed. Comparing WT with PBS, a total of 111 representative significant differential metabolites were cross-selected from univariate statistical significance (*p* < 0.05, FDR < 0.05) and multivariate VIP values (VIP > 1). These significant metabolites were further refined with the criteria of significant changes at the terminal stage (96 hpi, *p* < 0.05, FDR < 0.05, ratio > 2 or ratio < 0.5), followed by stricter analysis quality (%RSD < 10% in QCs). A total of 27 biomarker candidates were finally retained. They were designated the most representative metabolites, indicating infection-induced disturbances of the host. These candidates were subjected to receiver operating characteristic curves (ROCs) analysis to quantize their discrimination performance. The closer the AUC (area under the ROC curve) value approaches 1, the better the model provides discrimination potential. Then, candidates with the top AUC value, namely, dihydrosphingosine and FA 16:2, were assumed to be potential biomarkers (Fig. 5A,B, Fig. S5A,B). The combination of these two potential biomarkers achieved an AUC value of 0.889 in the discrimination of all WT samples (including different stages of WT samples) and PBS, and the sensitivity and specificity were 82.9% and 88.1%, respectively (Fig. 5C, Table S3).

These 27 representative infection features were further evaluated in the *clpV*-RNAi group. Comparing *clpV*-RNAi with WT, a total of 21 out of 27 candidates (77.8%) were validated with significant changes (*p* < 0.05 and FDR < 0.05). Satisfactory ROC results were also acquired in the application of those two potential biomarkers (Fig. S5C,D), resulting in an AUC value of 0.931 and sensitivity and specificity of 85.4% and 92.9%, respectively (Fig. 5D, Table S3). These analyses confirmed our previous finding that *clpV* is a key virulence gene during in vivo *P. plecoglossicida* infection. The combination of potential biomarkers dihydrosphingosine and FA 16:2 provided an effective model for discrimination of *P. plecoglossicida* infection.

## Discussion

*P. plecoglossicida* is the pathogen of ‘visceral white spot disease’ for marine teleosts, which can result in serious economic losses for aquaculture^1^. The *P. plecoglossicida-E. coioides* infection model has been confirmed to be a suitable animal model for studying the pathogenic mechanism of *P. plecoglossicida* in the laboratory^2,6–9^. Our previous gene expression studies revealed that *clpV* is a key virulence gene during *P. plecoglossicida* infection^2,3^. Compared with the counterparts infected by the wild-type strain, *E. coioides* injected with the *clpV*-RNAi strain exhibited a significant improvement in mortality and symptoms^2^. The histological examination confirmed the efficiency of *clpV* silencing on the improvement of infection symptoms (Fig. S1). To trace the interaction process between host and pathogen, a non-targeted metabolomics analysis was further performed in this study (Fig. 1A).

Global perturbations of *E. coioides* metabolome upon wild-type strain infection were clearly depicted by PCA (i.e., WT vs. PBS, Fig. 1D). The active changes could be noted by metabolic profiles from the early infection stage (6 hpi, Fig. 1E,F), confirming the efficacy of the current metabolomics study. Metabolic homeostasis showed increased disturbances across the course of the infection process, consistent with the increasingly severe symptoms of *E. coioides* (Fig. 1E,F). A decreased correlation between metabolites was also observed in WT when compared with control PBS (Fig. 3B). WT infection resulted in the most significant differences associated with amino acids, LPCs, and unsaturated FAs (Fig. 2A). Linoleic acid metabolism, arginine biosynthesis, alanine, aspartate and glutamate metabolism, glycerophospholipid metabolism, d-glutamine and d-glutamate metabolism, etc., were highly responsible for infection by the wild-type strain. The combination of potential biomarkers dihydrosphingosine and FA 16:2 provided an effective model for discrimination of *P. plecoglossicida* infection (Fig. 5, Table S3).

This metabolomics profiling highlighted the silencing of *P. plecoglossicida clpV* on improving infection. Metabolome changes associated with the influence of *clpV* were identified from the differences between *clpV*-RNAi and WT along the direction of the first principal component in the PCA score plot (i.e., *clpV*-RNAi vs. WT, Fig. 1D). Specifically, approximately 80% of significant differential metabolites identified by WT infection showed significant changes by *clpV* silencing (Fig. 1G). The coordination of metabolites was also reactivated in the *clpV*-RNAi group (Fig. 3B). Both the most enriched disturbed metabolites and metabolic pathways were similar when WT was compared with control PBS and *clpV*-RNAi. Comparing *clpV*-RNAi with WT, satisfactory ROC results were also acquired in the application of potential biomarkers dihydrosphingosine and FA 16:2 (Fig. 5, Table S3). This metabolomics results confirmed our findings regarding phenotype (incl. mortality, symptoms and histological examinations of *E. coioides*) and gene expression^2,3^, highlighting that *clpV* is a key virulence gene during in vivo *P. plecoglossicida* infection.

In this infection model, *P. plecoglossicida clpV* silencing greatly recovered the lipid metabolism of *E. coioides* (Fig. 3C). Although the improvement of amino acid metabolism was weaker than that of lipid metabolism after *clpV* silencing (Fig. 3C), the recovery of central points of competition between the host and pathogen, i.e., Arg & ornithine, glutamate (Glu) & glutamine (Gln), and Asn, could not be ignored.

### Amino acid metabolism

**Figure 6:**
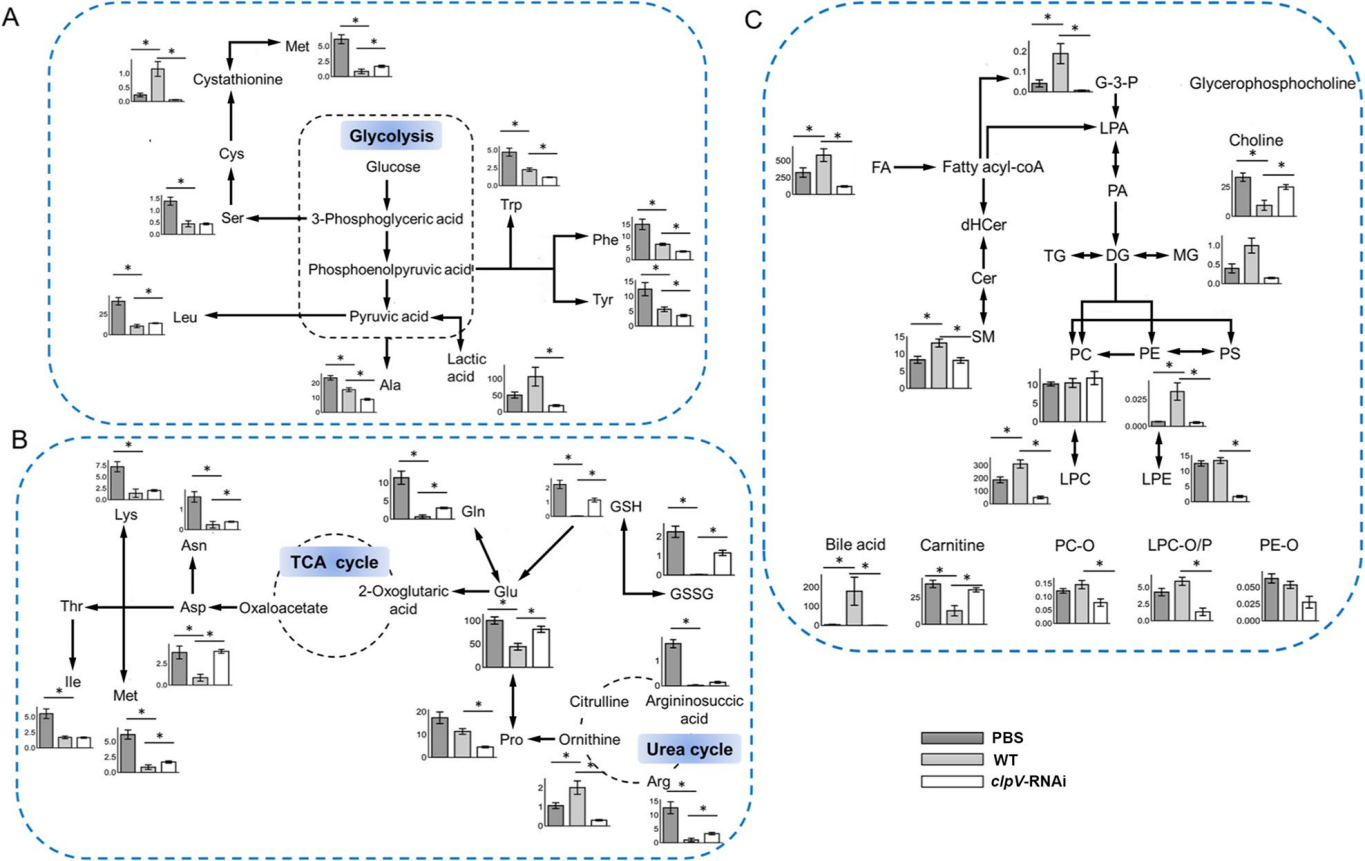
Profile of metabolite response at the terminal stage of 96 hpi. (**A,B**) Present the mapping of key pathways of amino acid metabolism. (**C**) Metabolism of lipids. All data are presented as the mean ± SE. The black asterisk indicates statistical significance (*p* < 0.05).

Our study revealed a decrease in most amino acids (incl. essential and nonessential amino acids) at the terminal infection stage (Fig. 6A,B). Amino acids affect the host’s physiology by serving as an energy source for cells, a basic substrate for protein synthesis and a regulator of cell signaling pathways^15^. Pathogens also show potent nutritional versatility in the assimilation of various amino acids that are present in their hosts^15^. Thus, the downregulation of amino acids after infection could potentially serve as a pathogenic strategy responsible for the competition of nutritional utilization by pathogens and the inhibition of host physiology.

#### Arg-ornithine metabolism

When compared with control PBS and *clpV*-RNAi, ornithine was observed as an exception with a significant increase upon WT infection, while its substrate Arg was significantly depleted in WT (Fig. 6B).

There are two predominant pathways for host Arg metabolism, i.e., via NO synthase (NOS) for the production of NO, and via arginase for the production of ornithine^15^. As an antimicrobial molecule, NO can protect the host against pathogens^17^. Conversely, ornithine can be used to produce polyamines (putrescine, spermidine, and spermine), which are essential for the proliferation of pathogens^18^. Arginase competes with NOS for Arg depletion during infections^13,15^. Then, the metabolic signature of ornithine and substrate Arg discovered in this study might suggest the promotion of ornithine synthesis, attributed to the extensive competition for Arg utilization between the host and pathogen. Pathogens alter the metabolic flux of host Arg from producing antimicrobial NO to producing polyamines by increasing the expression of arginase to facilitate their growth. Other mechanisms, including Arg transport and the Arg deiminase (ADI) pathway to deplete Arg and/or divert Arg away from host cells that produce NO, have also been identified in previous studies of host–pathogen interactions^19^. Moreover, in the *clpV*-RNAi group, the upregulation of Arg and downregulation of ornithine confirmed the efficacy of *clpV* silencing on the reduction of *P. plecoglossicida* virulence.

#### Glu metabolism (GM)

Glu is crucial for pathogens due to its involvement in a wide range of metabolic processes^15,20^. In addition to being transported from the extracellular milieu, Glu can be enzymatically generated by cytosolic Gln (via glutaminase) and aspartate (Asp) (via aspartate transpeptidase)^21^. Furthermore, Glu is the precursor to synthesize glutathione (GSH)^20,21^.

In this study, GM-related metabolites, including Glu, Gln, Asp, GSH and glutathione disulfide (GSSG), were significantly decreased upon WT infection (Fig. 6B). Glu is an important nutritional source for pathogens^20^. In WT, the depletion of Glu and its precursors Gln and Asp might be attributed to the direct competition between pathogens and host cells for the nutrient pools. In addition, as an important product of Glu, GSH contributes to oxidative stress defense by playing a critical role as a redox buffer to detoxify noxious oxygen species^21^. The downregulation of GSH and Glu could attenuate the antioxidant capacity of the host tissue.

In *clpV*-RNAi, GM-related metabolites were significantly recovered (Fig. 6). The silencing of the key virulence gene *clpV* weakens the pathogenic competitive utilization of GM-related nutrient substrates and facilitates the improvement of the oxidative stress defense of the host.

#### Asn metabolism

Asn is another central point of interaction^13,15^, and was significantly decreased upon infection with a slight rebound in *clpV*-RNAi.

Asn has diverse roles in bacterial physiology. Asn represents an important nitrogen source for pathogens and is also essential for an intracellular pathogen to resist acidic stress in a phagosome by serving as the substrate for the production of the weak base ammonia^22^. In addition to its role in pathogen survival and infection, Asn is necessary for lymphocyte activation and protective immunity of the host^23^. Asn may affect T cell functions via mTORC1 which is critically important for T cell activation and differentiation^15^. Thus, the pathogen could inhibit the immune response of the host by inducing starvation of Asn in host cells. The depletion of Asn could be responsible for the inhibition of immune responses in the host.

### FA and LPC metabolism

Defined as the top significant differential metabolite classes in enrichment analyses, FAs and LPCs had a greater response and a higher degree of unsaturation in WT compared with the control PBS and *clpV*-RNAi (Fig. 6C). Of note, we found a significant positive correlation between the increase in the WT to PBS ratio and the number of double bonds (*p* < 0.05, Fig. 4A,B). Their increase in the WT to *clpV-*RNAi ratio had a similar significant positive correlation with the degree of unsaturation (*p* < 0.05, Fig. 4A,B).

The modulation of lipids in content and composition by virulent pathogens is an effective strategy to facilitate their invasion and propagation in host cells^10,11^. FAs and lyso-compounds tend to be used as building blocks for this complex lipidome modulation^11^. As key players in inducing inflammation, lipid mediators of prostaglandins and leukotrienes are synthesized from phospholipid-derived polyunsaturated FAs (PUFAs)^24^. Phospholipase, hydrolyzes phospholipids into fatty acids and other lipophilic substances (e.g., lyso-phospholipids), leading to membrane dysfunction or even disruption of the cell^11^. Then, cyclooxygenase COX-2 converts the PUFA of arachidonic acid to prostaglandin endoperoxide H2^11^. To promote the synthesis of prostaglandins and leukotrienes, both Gram-negative and Gram-positive bacteria can trigger signal transduction pathways that enhance the activities of phospholipase and/or cyclooxygenase COX-2 in targeted cells^25^. *Salmonella* can deliver the effector protein SpiC to the cytosol of infected macrophages to alter host cell signaling and then promote an immunosuppressive phenotype that impairs bacterial killing^26^. Similar mechanisms are used by *Pseudomonas* to promote prostaglandin production for their own benefit^11^. Thus, the changes in those lipids with a complex structure of polyunsaturated hydrocarbon chains produced from the pools of FAs and LPCs confirmed the inflammation induction in WT and the significant improvement of inflammation in *clpV*-RNAi.

Taken together, as the direct link of the metabolome with cellular phenotype, the cellular metabolome would be an attractive target for pathogens to modulate host cell processes to facilitate their survival and replication^15,27^. Metabolic crosstalk between host and pathogen profoundly shapes the pathogenesis of an infection^15^. Currently, research in the area of cell biology has yielded a series of findings regarding *P. plecoglossicida* infection^2,3,6–9^; however, the analysis of metabolic crosstalk between host and pathogen is limited and thereby has gained interest recently. Our metabolomics study revealed global metabolic changes associated with wild-type strain infection, indicating key disturbances in host nutritional utilization and immune responses. From this perspective of the metabolome, our study highlighted the efficiency of the key virulence gene *clpV*, consistent with our previous gene expression discoveries. Further research will be performed involving a more comprehensive lipidomics study.

In this study, a *P. plecoglossicida* strain (NZBD9) and the economic fish *E. coioides* were investigated as an attractive model system to characterize pathogen-induced host metabolic remodeling over the course of infection using nontargeted metabolomics approach. This comprehensive metabolomics study revealed how strains shape host metabolism to support their survival and replication and highlighted the potential of the virulence gene *clpV* in the treatment of an infection. Then, this study would expand our understanding of biochemical mechanism for host–pathogen interactions and provide novel clues for therapeutic approaches to manipulate and prevent progression of an infection in aquaculture.

## Materials and methods

### Bacterial strains

The wild-type *P. plecoglossicida* strain (NZBD9) was isolated from the spleen of naturally infected *Larimichthys crocea*^28^. According to previous methods with minor modification^2,29^, the *P. plecoglossicida* RNAi strain with optimal silencing efficiency targeting *clpV* was constructed. Details of the wild-type strain and *clpV*-RNAi strain are provided in our previously published studies^2^.

### *E. coioides* infection and sampling

All *E. coioides* (about 100 g) were obtained from Zhangzhou (China). The protocol of the fish experiment was approved by the Animal Ethics Committee of Jimei University (Acceptance NO. JMULAC201159, Xiamen, China), complying with the ‘Guide for the Care and Use of Laboratory Animals’ set by the National Institutes of Health. The study is also in accordance with ARRIVE guidelines (https://arriveguidelines.org).

The *P. plecoglossicida*–*E. coioides* infection model was established as described in our previous reports^2,7–9^. The scheme of this fish experiment is provided in Fig. 1A. Briefly, after 1 week of adaptive inhabitation at 18 °C under specific pathogen-free laboratory conditions, all size-matched *E. coioides* were randomly divided into three groups: PBS control group (PBS group), wild-type strain infection group (WT group) and *clpV*-RNAi strain infection group (*clpV*-RNAi group). *E. coioides* of the infection group were intrapleurally injected with 10^5^ colony-forming units per fish (cfu/fish) of *P. plecoglossicida* (wild-type strain or *clpV*-RNAi strain), while control *E. coioides* received an equivalent volume of PBS. The temperature of the water was maintained at 18 ± 1 °C during the infection experiment. The spleens of *E. coioides* were collected at 6, 12, 24, 48, 72 and 96 hpi. Seven independent biological replicates were prepared for each group at each time point. Finally, all 126 spleen specimens from 6 time points were stored immediately at − 80 °C.

### Non-targeted metabolomics analysis

The metabolite extract (cf. Supplement) from each biological replicate was analyzed to conduct a non-targeted metabolomics study using an ACQUITY UPLC system (Waters, USA) coupled with Q-Exactive HF MS (Thermo Fisher Scientific, USA). Details of the metabolomics analysis including sample preparation, equipment, and methods are provided in the Supporting Information.

QC samples were obtained from pooled metabolic extracts and prepared as real samples^30^. These QC samples were analyzed every 10 injections during the entire run to monitor the robustness of the analysis.

### Data processing and statistics

Approximately 2000 metabolite standards were preanalyzed by our collaborator to develop an in-house database^31^. This in-house database was used for peak identification based on accurate m/z, MS/MS fragmentation patterns, and retention time^31^. The original intensities of metabolites were normalized to the weight of spleen tissue, followed by the intensity of internal standards to eliminate systematic bias. Then, this processed dataset was employed for subsequent statistical analysis. Details of the data processing and statistics are provided in the Supporting Information.

## Supplementary Information

Supplementary Information.

Supplementary Table S1.

Supplementary Table S2.

Supplementary Table S3.

## Data Availability

The datasets generated and/or analysed during the current study are available in the Metabolomics Workbench (https://www.metabolomicsworkbench.org/) repository (Data Track ID 3186).
