# Supplementary material for: Metabolomics insights into the interaction between Pseudomonas plecoglossicida and Epinephelus coioides
Source: Sci Rep. 2022 Aug 3;12:13309. doi: 10.1038/s41598-022-17387-6 (PMC9349296; doi:10.1038/s41598-022-17387-6)
Supplement: Supplementary file 1 — Supplementary Information. [file 41598_2022_17387_MOESM1_ESM.docx]

**Supporting Information**

**Metabolomics Insights into the Interaction between *Pseudomonas plecoglossicida* and** ***Epinephelus coioides***

Jun Zeng^1,3,#,*^, Zhiqiang Yang^1,#^, Yue Zhong^1^, Yingli Zheng^1^, Jingwen Hao^1^, Gang Luo^2^, Qingpi Yan^2,*^

^1^ College of Ocean Food and Biological Engineering, Jimei University, Xiamen, 361021, China

^2^ Fisheries College, Jimei University, Xiamen, 361021, China

^3^ Xiamen Key Laboratory of Marine Functional Food, Xiamen 361021, China

^#^: Equal contribution

*: Corresponding authors

Qingpi Yan, Tel.: 86-592-6183028. E-mail: yanqp@jmu.edu.cn

Jun Zeng, Tel.: 86-592-6181487. E-mail: junzeng@jmu.edu.cn

**Content:**

1. Materials and methods of **Materials and Chemicals**

2. Materials and methods of **Sample Preparation**

3. Materials and methods of **Non-targeted Metabolomics Analysis**

4. Materials and methods of **Data Processing and Statistics**

5. Results of **Analytical Performance of Metabolomics Profiling**

6. Table S1. Information of metabolites.

7. Table S2. Statistical results of representative differential metabolites (*p* < 0.05, FDR < 0.05 and VIP > 1).

8. Table S3. The result of ROC analysis for the evaluation of discrimination potential.

9. Figure S1. Typical microphotographs of spleen tissues taken at the terminal stage of infection (hematoxylin and eosin (H&E), original magnification × 400).

10. Figure S2. Analytical performance of metabolomics profiling.

11. Figure S3. PLS-DA score plot.

12. Figure S4. Histograms of VIP values with jack-knifed confidence intervals.

13. Figure S5. Evaluation of potential biomarkers.

**Materials and methods**

**Materials and Chemicals**

Methanol, acetonitrile and isopropanol were purchased from Merk (Germany). Tert-butyl methyl ether (MTBE) and formic acid were purchased from Sigma-Aldrich (USA). All reagents were high performance liquid chromatography (HPLC)-grade. Ammonium bicarbonate was purchased from Sigma-Aldrich. Stable isotope standards of phenylalanine (Phe)-d5, tryptophan (Trp)-d5, acetylcarnitine (carnitine C2)-d3, cholic acid (CA)-d4, chenodeoxycholic acid (CDCA)-d4, palmitic acid (fatty acid (FA) 16:0)-d3 and stearic acid (FA C18:0)-d3 were purchased from Cambridge Isotope Laboratories (USA) and Sigma-Aldrich. Other standards of lysophospatidylcholine (LPC) (19:0), sphingomyelin (SM) (d18:1/12:0) were obtained from Avanti Polar Lipids (USA). Ultrapure water was prepared by a Millipore Milli-Q system (USA).

**Sample** **Preparation**

The deep-frozen spleen tissue in each replicate was accurately weighed (about 15 mg) and then transferred to an Eppendorf tube. Each sample was first spiked with 300 μL of methanol containing internal standards (0.54 μg/mL of Phe-d5, 0.5 μg/mL of Trp-d5, 0.28 μg/mL of carnitine C2-d3, 0.27 μg/mL of CA-d4, 0.1 μg/mL of CDCA-d4, 1 μg/mL of FA 16:0-d3, 1 μg/mL of FA 18:0-d3, 1.08 μg/mL of LPC 19:0, 0.87 μg/mL of SM (d18:1/12:0)) and then homogenized using a bead-based homogenizer (Tissuelyser-24, Shanghai Jingxin Industrial Development Co., Ltd, China) at 65 Hz for 2.5 min. The homogenate was mixed with 1 mL of MTBE, and thoroughly vortexed for 30 min. Then, 300 μL of Milli-Q water was added to the tube, followed by vortexing for 1 min and centrifugation for 10 min to form a two-phase system (12,000 rpm, 4 ℃). Each specimen was prepared by mixing 250 μL of the top layer plus 150 μL of the bottom layer accurately and then vacuum-dried in a Speedvac concentrator (Thermo Scientific, USA). These specimens were stored at -80 °C until dissolved for subsequent analysis.

**Non-targeted Metabolomics Analysis**

ACQUITY ultra-performance liquid chromatography (UPLC, Waters, USA) coupled with Q-Exactive HF mass spectrometry (Thermo Fisher Scientific, USA) was performed in this study. Non-targeted metabolomics profiling was acquired in both positive and negative analysis modes.

For UPLC separation, a reversed phase BEH C8 column (2.1 × 100 mm, 1.7 μm) was used for the positive mode. Mobile phase A was water and B was acetonitrile, both containing 0.1% (v/v) formic acid. A 30 min of elution gradient was as follows: 5% B was firstly maintained for 1 min, then linearly increased to 100% B from 1 to 24 min, and maintained at 100% B for 4 min, followed by the decrease to 5% B in 0.1 min and equilibration until the next injection. In the negative mode, metabolic extracts were separated using a HSS T3 column (2.1 × 100 mm, 1.8 μm) (Waters, USA). Mobile phase A was water and B was methanol/water (95:5, v/v), both containing 6.5 mM ammonium bicarbonate. A 25 min of elution gradient was as follows: 2% B was firstly maintained for 1 min, then linearly increased to 100% B from 1 to 18 min, and maintained at 100% B for 4 min, followed by the decrease to 5% B in 0.1 min and equilibration until the next injection. The flow rate of UPLC was 0.35 mL/min for both positive and negative modes, and the temperature of column and sample tray were set at 50 and 6 ℃, respectively. Injection volume was set at 5 μL.

The MS acquisition was operated with full scan MS for profiling and data-dependent MS/MS (ddMS2) for identification. When heated electrospray (HESI) was applied in the positive ion mode, HESI source setting of spray voltage, capillary temperature, sheath gas flow rate and aux gas flow rate were 3.5 kV, 320 ℃, 50 arb and 15 arb, respectively. High resolution MS and MS/MS (ddMS2) acquisition was applied with normalized collision energy (NCE) of 25 and 35, TopN (N, the number of top most abundant ions for fragmentation) of 10, resolution of 140, 000 for full scan MS and 35, 000 for ddMS2, and scan range of 80-1200 m/z. When HESI was performed in the negative ion mode, most parameters were identical to those used in the positive mode, except that the spray voltage were reset at 3.0 kV.

**Data Processing and Statistics**

Raw metabolomics data obtained from UPLC-MS analysis were processed using XCMS software (https://xcmsonline.scripps.edu/) to export a peak table containing peak features (m/z and retention time) and intensities. Approximately 2000 metabolite standards were preanalyzed by our collaborator to develop an in-house database[1]. This in-house database was used for peak identification based on accurate m/z, MS/MS fragmentation patterns, and retention time[1]. Furthermore, quantitative levels of these identified metabolites were reassessed by high-resolution EIC (extracted ion chromatogram) using Trace Finder software (Thermo, USA) with a m/z tolerance of ± 10 ppm and a retention time extraction window of ± 15 s. Noise removal was conducted to reduce errors. The original intensities of metabolites were normalized to the weight of spleen tissue, followed by the intensity of internal standards to eliminate systematic bias. Then, this processed dataset was employed for subsequent statistical analysis.

Multivariate pattern recognition was performed by SIMCA-P software (Umetrics, Sweden) with unit variance (UV) scaling. Multi Experiment Viewer (MeV) software (open-source genomic analysis software) and an in-house developed MATLAB program (The MathWorks, USA) were used for the univariate analysis. The univariate statistical significance was assessed by two-way analysis of variance (ANOVA), Wilcoxon Mann-Whitney test, and false discovery rate (FDR) correction (Benjamini-Hochberg method). The *p* values by two-way ANOVA evaluating the infection effect and gene change were used in this study to determine those significant differential metabolites. A heatmap based on hierarchical cluster analysis (HCA) was also generated by MeV to visualize the relative levels and relationships of metabolites. An open-source software platform of Cytoscape was applied to analyze the metabolic correlation network. Receiver operating characteristic curves (ROCs) were made by SPSS Statistics software (SPSS Inc., USA). Pathway enrichment was conducted by the pathway analysis module of MetaboAnalyst (http://www.metaboanalyst.ca). Those important metabolic pathways were further analyzed based on the references of Kyoto Encyclopedia of Genes and Genomes (KEGG) and Human Metabolome Database (HMDB).

**Results**

**Analytical Performance of Metabolomics Profiling**

The repeatability of QC samples was evaluated to ensure the reliability of metabolomics analysis. As illustrated in score plots by principal component analyses (PCA), QC samples from positive and negative datasets cluster closely in each analysis mode (Figure S2A and B), and all QC samples were within two times of the standard deviation (SD) (Figure S2C and D). Additionally, metabolites with the relative standard deviation (RSD) less than 30% in QC samples accounted for 98.85% and 97.54% of the total metabolite number for positive and negative modes, respectively (Figure S2E and F). These results demonstrated a desirable analytical performance of present metabolomics study.

Datasets from positive and negative analysis modes were combined for subsequent statistical analysis. To improve the quality of data analysis, metabolites with the RSD higher than 30% in all QCs were removed from the dataset prior to subsequent study.


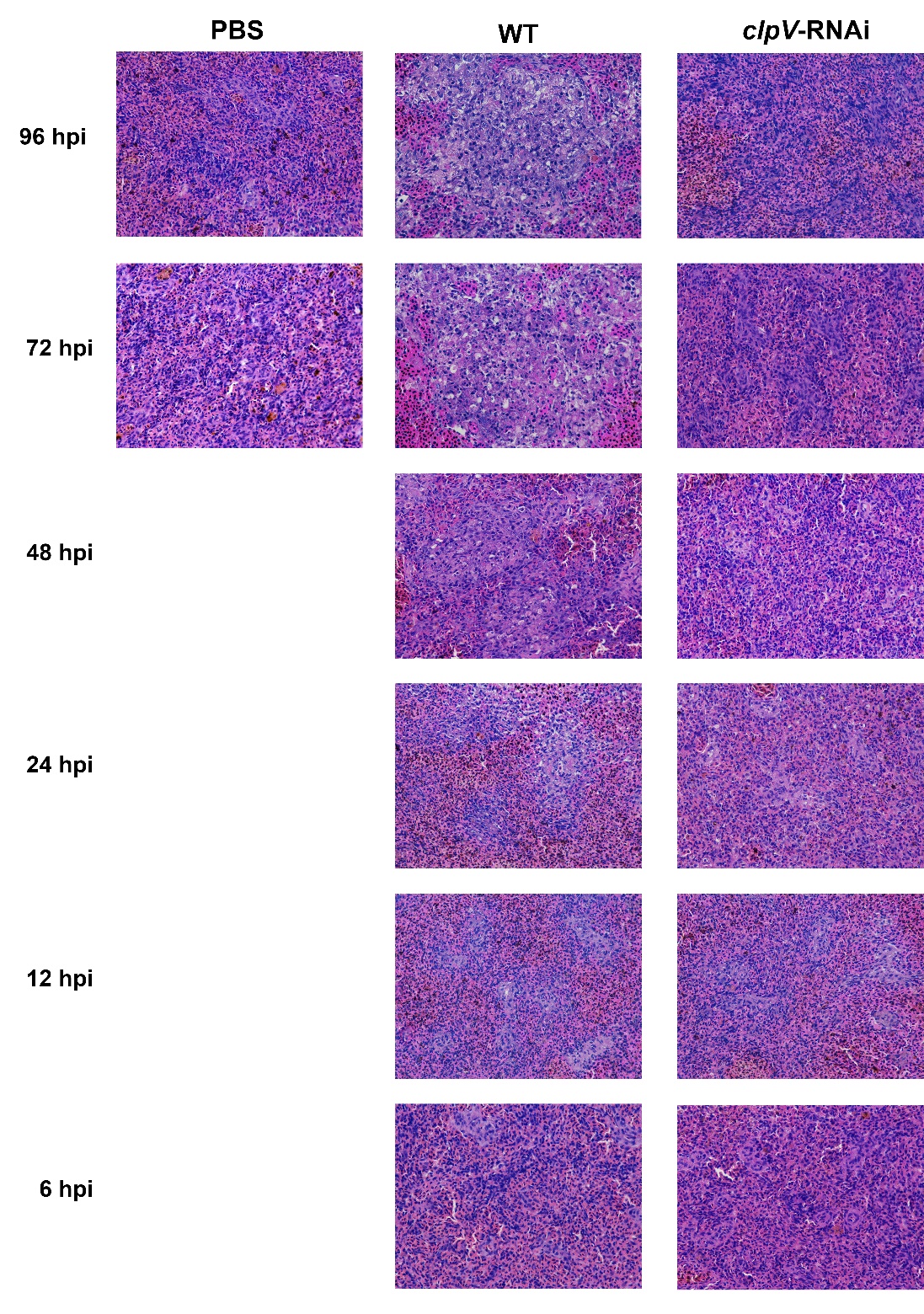


**Figure S1. Typical microphotographs of spleen tissues taken at the terminal stage of infection (hematoxylin and eosin (H&E), original magnification × 400).** PBS denotes the PBS control group, WT denotes the wild-type strain infection group, and *clpV-*RNAi denotes the *clpV*-RNAi strain infection group.

**
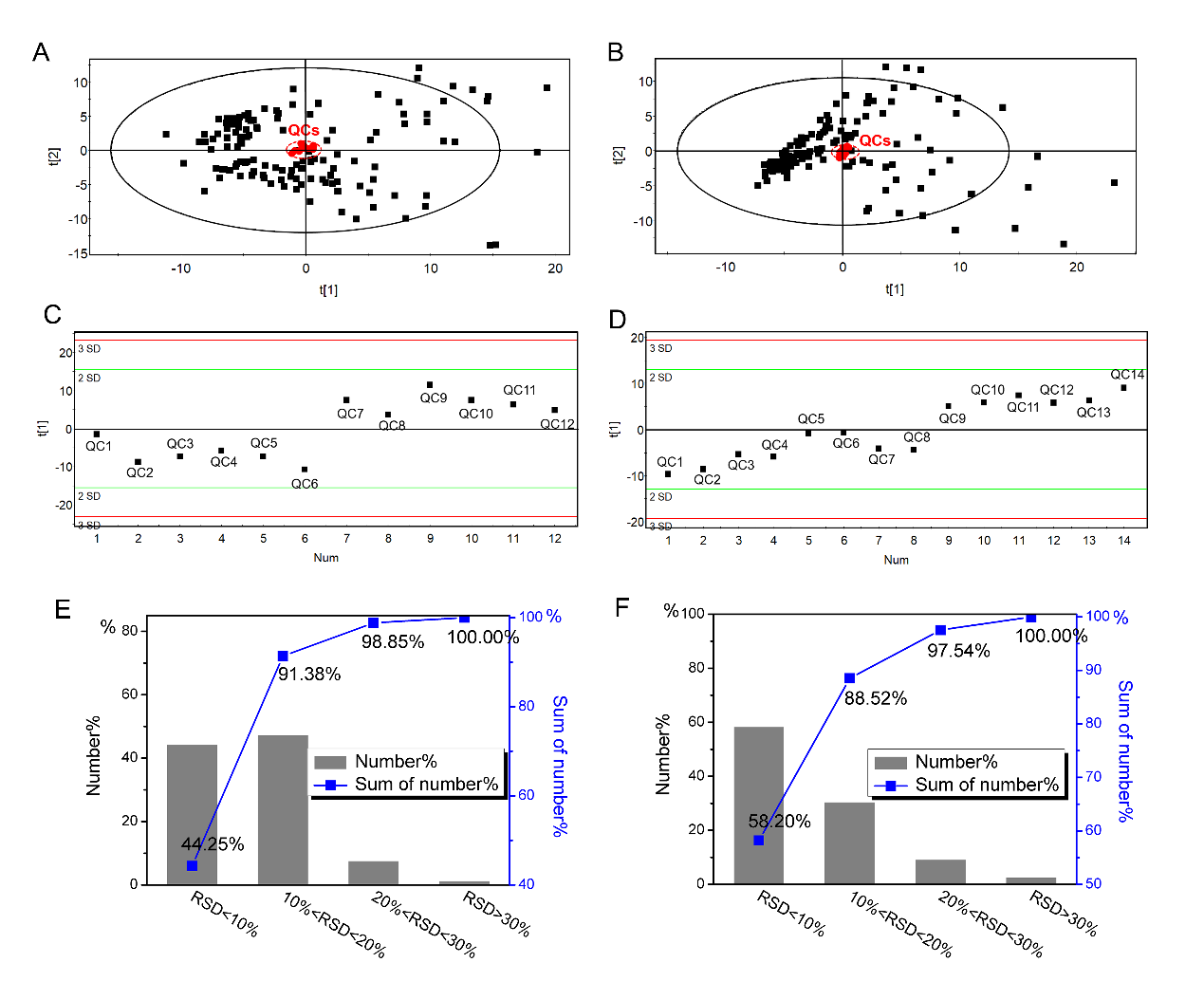
**

**Figure S2. Analytical performance of metabolomics profiling.** (A) and (B) are PCA score plots for all samples from positive and negative analysis mode, respectively. (C) and (D) are PCA score plots for QC samples from positive and negative analysis mode, respectively. (E) and (F) indicate the disturbance of %RSD for metabolites among all QC samples from positive and negative analysis mode, respectively.

**
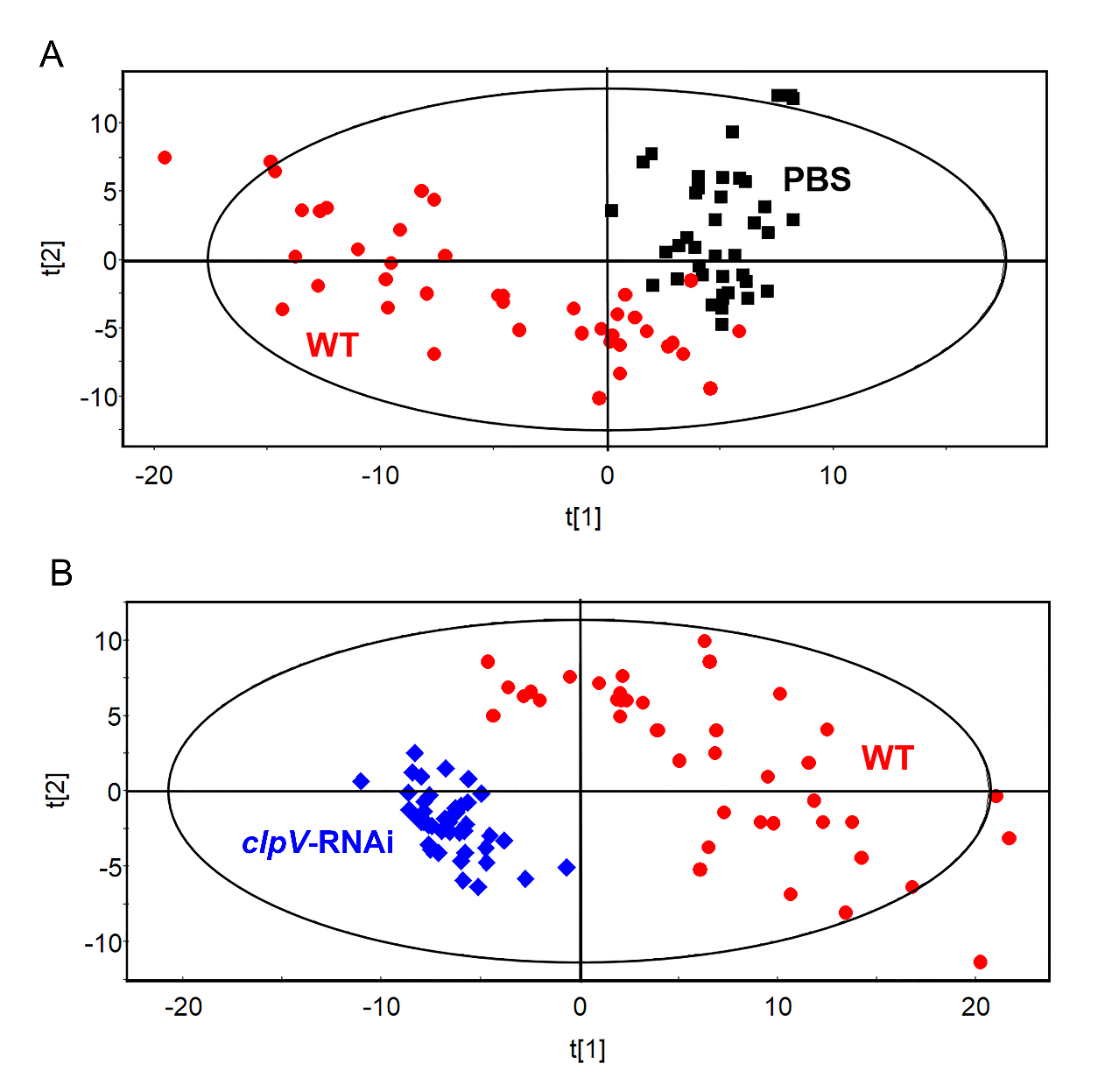
**

**Figure S3. PLS-DA score plot.** (A) and (B) are developed from two PLS-DA models (i.e., PBS vs. WT and *clpV*-RNAi vs. WT).

**
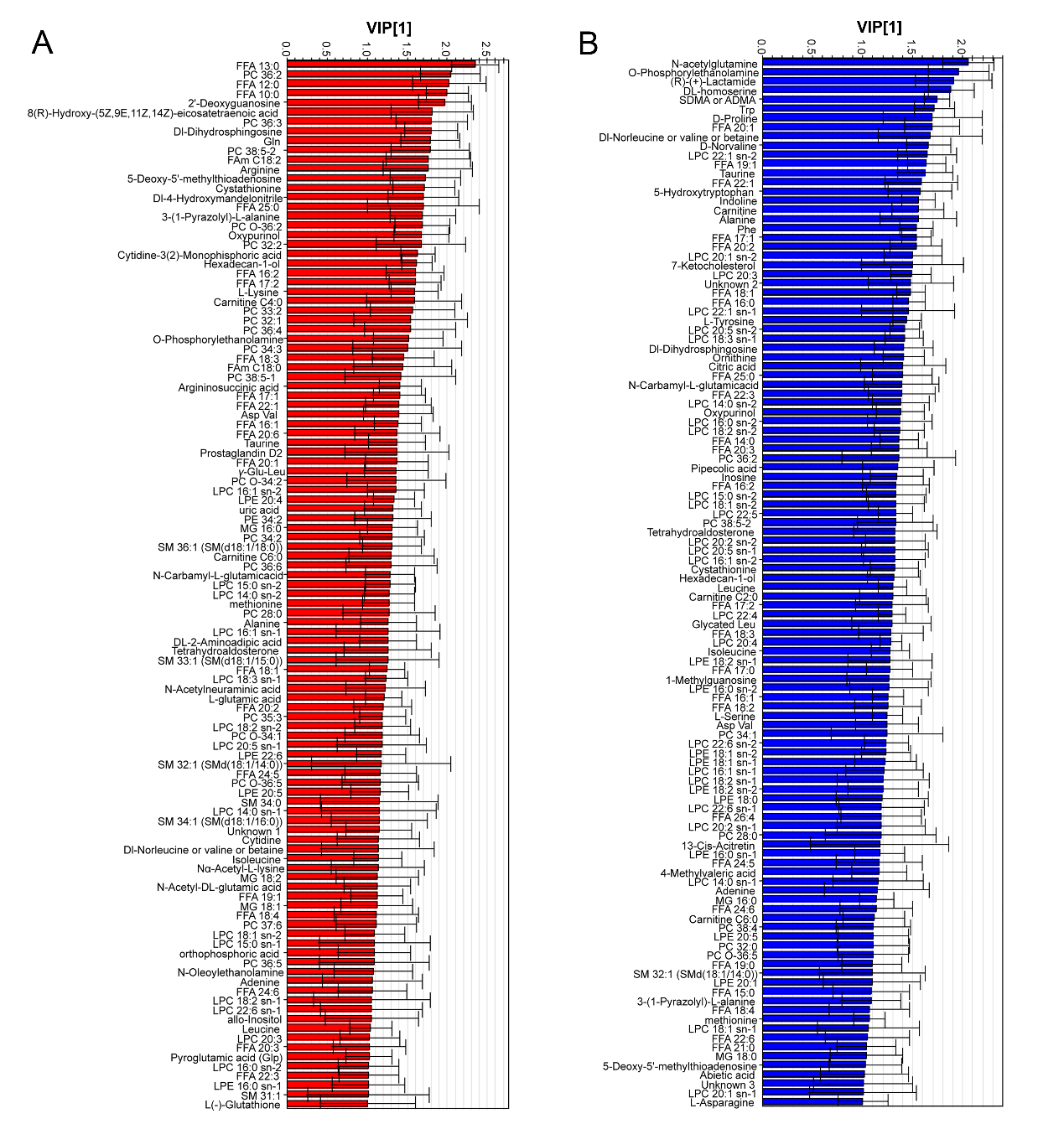
**

**Figure S4. Histograms of VIP values with jack-knifed confidence intervals.** (A) and (B) present the VIP values that were obtained from the first principal component of two PLS-DA models (i.e., PBS vs. WT and *clpV*-RNAi vs. WT), respectively. Lipids with VIP > 1 were listed in the plots.

**
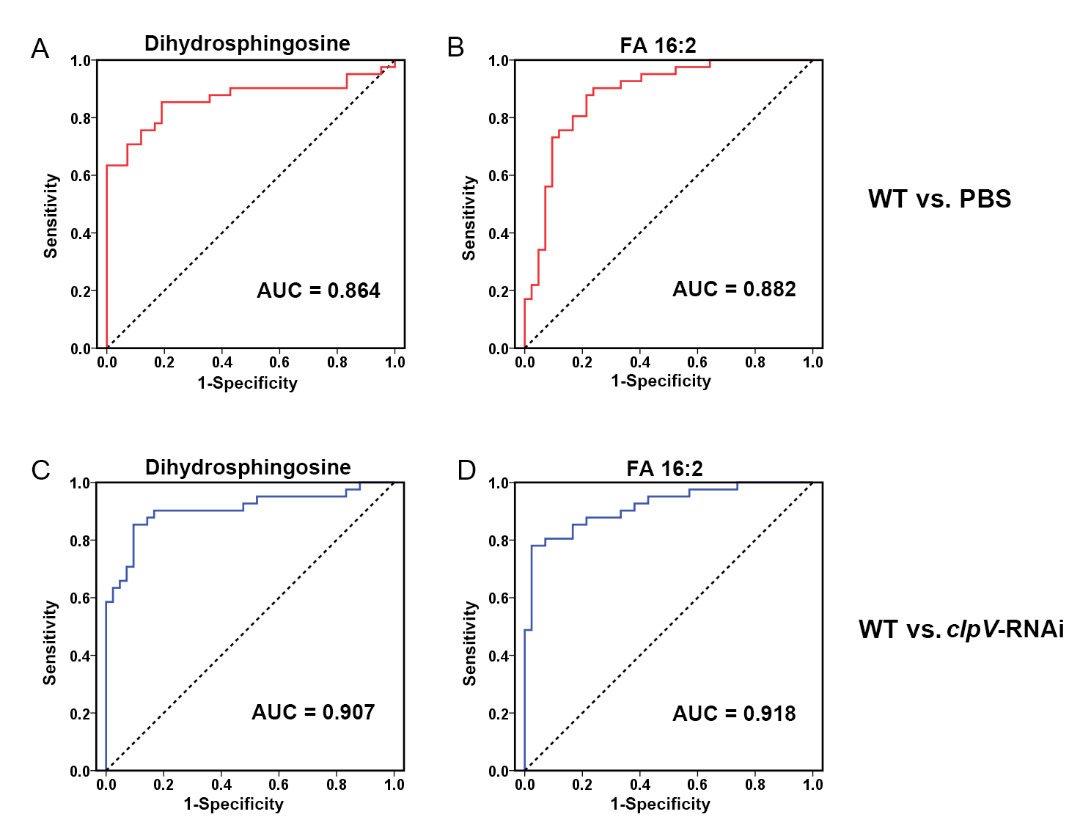
**

**Figure S5. Evaluation of potential biomarkers.** (A)-(D) are ROC curves for potential biomarkers. Diagnostic potential was evaluated based on binary logistic regression.

**REFERENCES**

1. Zhao XJ, Zeng ZD, Chen AM, et al. Comprehensive Strategy to Construct In-House Database for Accurate and Batch Identification of Small Molecular Metabolites [Article]. Anal Chem. 2018 Jun;90(12):7635-7643.
